# Fever and Cholera Precautions

**Published:** 1888-08-18

**Authors:** George Buchanan

**Affiliations:** Principal Medical Officer to the Local Government Board.


### Fever and Cholera Precautions.

By Dr. George Buchanan, F.R.S., Principal Medical Officer to the Local Government Board.

An important memorandum on the proceedings which are advisable in places attacked or threatened with epidemic disease, which has been drawn up by Dr. George Buchanan, F.R.S., has been issued to sanitary authorities by the Local Government Board. In this memorandum it is stated that wherever there is prevalence or threatening of cholera, diphtheria, fever, or any other epidemic disease, it is of more than common importance that the statutory powers conferred upon sanitary authorities for the protection of the public health should be well exercised by those authorities, acting with the advice of their medical officers of health.

Proper precautions are equally requisite for all classes of society. But it is chiefly with regard to the poorer population, therefore chiefly in the courts and alleys of towns, and at the labourers' cottages of country districts, that local authorities are called upon to exercise vigilance and to proffer information and advice. Common lodging-houses and houses which are sub-let in several small holdings always require particular attention.

Wherever there is accumulation, stink, or soakage of house refuse or of other decaying animal or vegetable matter the nuisance should as promptly as possible be abated, and precaution should be taken not to let it recur. Especially examination should be made as to the efficient working of sewers and drains, and any defect therein and any nuisance therefrom, or from any foul ditches or ponds, should be got rid of without delay. The ventilation of sewers, the ventilation and trapping of house drains, and the disconnexion of cistern overflows and sink pipes from drains, should be carefully seen to. The scavenging of the district, and the state of receptacles for excrement and of dust-bins will require close attention. In slaughter-houses, and wherever animals are kept, strict cleanliness should be enforced.

In the removal of filth during periods of epidemic disease it is commonly necessary to employ chemical agents for reducing or removing the offence and harm which may be involved in the disturbance of the filth. In the removal of privy contents these agents are more particularly wanted if the disease in question be cholera or enteric fever. The chemical agent should be used liberally over all exposed surfaces from which filth has been removed. Unpaved earth close to dwellings, if it be sodden with slops or filth, ought to be treated in the same way.

Sources of water supply should be well examined. Water from sources which can be in any way tainted by animal or vegetable refuse, especially those into which there may be any leakage or filtration from sewers, drains, cesspools, or foul ditches, ought no longer to be drunk. Above all, where the disease is cholera, diarrhœa, or enteric fever, it is essential that no impure water be drunk.

The liability of leaky pipes to act as land drains and to receive foul matters as well as land drainage through their leaks is not to be overlooked. And such leaky pipes, running full of water with considerable velocity, are liable to receive, by lateral insuction at their points of leakage, external matters that may be dangerous. This latter fact is not recognised so generally as it should be, and ignorance of it has probably baffled many inquiries in cases where water services have in truth been the means of spreading disease.

If, unfortunately, the only water which for a time can be got should be open to suspicion of dangerous organic impurity, it ought at least to be boiled before it is used for drinking, but then not to be drunk later than 24 hours after it has been boiled. Filtering of the ordinary kind cannot by itself be trusted to purify water. It cannot be too distinctly understood that dangerous qualities of water are not obviated by the addition of wine or spirits. When there appears any probable relation between the distribution of disease and of milk supplies, the cleanliness of dairies, the purity of the water used in them, the health of the persons employed about them, and the health of the cows that furnish milk should always be carefully investigated. Even apart from any apprehension of milk being concerned in a particular outbreak of disease, it is desirable that English people should adopt the custom, which is always followed in some Continental countries, of boiling all milk at once upon its reception into a house.

The washing and lime-whiting of uncleanly premises, especially of such as are densely occupied, should be pressed with all practicable despatch.

Overcrowding should be prevented. Especially where disease has begun, the sick room should, as far as possible, be free from persons who are not of use to the patient.

Ample ventilation should be enforced. It should be seen that windows are made to open, and that they are sufficiently opened. Especially where any kind of infective fever has begun, it is essential, both for patients and for persons who are about them, that the sick room and the sick house be constantly traversed by streams of fresh air.

The cleanliest domestic habits should be enjoined. Refuse matters should be speedily removed or destroyed; and things which have to be disinfected or cleansed should always be disinfected or cleansed without delay.

Special precautions of cleanliness and disinfection are necessary with regard to infective matters discharged from the bodies of the sick. Among discharges which it is proper to treat as infective are those which come in cases of smallpox and scarlatina from the affected skin; in cases of cholera and enteric fever from the intestinal canal; in cases of diphtheria and scarlatina from the nose and throat; likewise in cases of any eruptive or other epidemic fever, the general exhalations of the sick. The caution which is necessary with regard to such matters must, of course, extend to whatever is imbued with them; so that bedding, clothing, towels, handkerchiefs, and other articles which have been in use by the sick may not become sources of mischief, either in the house to which they belong, or in houses to which they are conveyed. So far as articles of this class can be replaced by rags or things of small value, it is best to use such things and burn them when they are soiled. Otherwise clothing and infected articles should be subjected to the disinfectant of the sick room or be removed for disinfection by heat.

In enteric fever and cholera the evacuation should be regarded as capable of communicating an infectious quality to any nightsoil with which they are mingled in privies, drains, or cesspools; and after such disinfection of the mass is practicable, they should be disposed of without delay and under the safest conditions that local circumstances permit. They should not be thrown into any fixed privy receptacle, and above all they must never be cast where they can run or soak into sources of drinking-water.

All reasonable care should be taken not to allow infective disease to spread by the unnecessary association of sick with healthy persons. This care is requisite, not only with regard to the sick house, but likewise with regard to schools and other establishments wherein members of many different households are accustomed to meet.

If disease begins in houses where the sick person cannot be properly accommodated and tended, medical advice should be taken as to the propriety of removing him to an infirmary or hospital. Every sanitary authority should have in readiness a hospital for the reception of such cases.

Where dangerous conditions of residence cannot be promptly remedied, it will be best that the inmates, while unattacked by disease, remove to some safer lodging.

Privation, as predisposing to disease, may require special measures of relief.

In certain cases special medical arrangements are necessary. For instance, as cases of cholera in this country often begin somewhat gradually in the comparatively tractable form of what is called "premonitory diarrhœa," it is essential that, where cholera has appeared, arrangements should be made for affording medical relief without delay to persons attacked, even slightly, with looseness of bowels. So, again, where smallpox is the prevailing disease, it is essential that all unvaccinated persons (unless they previously have had smallpox) should very promptly be vaccinated; and that revaccination should be performed in cases properly requiring it.

It is always to be desired that the people should, as far as possible, know what real precautions they can take against the disease which threatens them, what vigilance is needful with regard to its early symptoms, and what (if any) special arrangements have been made for giving medical assistance within the district. For the purpose of such information printed hand-bills or placards may usefully be employed, and in cases where danger is great house-to-house visitation by discreet and competent persons may be of the utmost service, both in quieting unreasonable alarm and in leading or assisting the less educated and the destitute parts of the population to do what is needful for safety.

The present memorandum relates to occasions of emergency. Therefore the measures suggested in it are essentially of an extemporaneous kind; and permanent provisions for securing the public health have, in express terms, been but little insisted on. It it to be remembered, however, that, in proportion as a district is habitually well cared for by its sanitary authority, the more formidable emergencies of epidemic disease are not likely to arise in it.

